# Spontaneous Otogenic Pneumocephalus due to Frequent Plane Travelling

**DOI:** 10.1155/2019/8768506

**Published:** 2019-03-19

**Authors:** Katherine Pollaers, Jafri Kuthubutheen

**Affiliations:** Ear, Nose and Throat Department, Royal Perth Hospital, 197 Wellington St Perth, Western Australia 6000, Australia

## Abstract

A 31-year-old male presented with a history of chronic right-sided facial and mastoid tip pain with associated tinnitus and hearing loss. These symptoms were aggravated by the regular aeroplane trips he made to work as a “fly-in, fly-out” worker in regional Australia. Imaging revealed significant pneumocephalus secondary to mastoid air cell defects, which were repaired via a transmastoid approach. This is the fourth case of spontaneous otogenic pneumocephalus associated with air travel at altitude reported in the literature. This case is remarkable for the chronic nature of the symptoms, which were aggravated by the patient's regular aeroplane travel. This has implications for occupations which require frequent flying in those patients who may be at risk.

## 1. Introduction

A pneumocephalus describes the clinical entity of intracranial free gas. Spontaneous otogenic pneumocephalus is a rare clinical entity, with only a handful of reported cases. In the case of spontaneous otogenic pneumocephalus, the source of abnormal intracranial air is felt to be hyperpnuematisation of the temporal bone. The manifestations are varied from asymptomatic to presenting an acute neurosurgical emergency. The typical presentation of otogenic pneumocephalus has not been delineated owing to the rarity of the clinical entity. We present the fourth case of spontaneous otogenic pneumocephalus associated with air travel at altitude reported in the literature. This case is remarkable for the chronic nature of the symptoms, which were aggravated by the patient's regular aeroplane travel. This has implications for occupations which require frequent flying in those patients who may be at risk.

## 2. Case Presentation

A 31-year-old male presented to an outpatient otolaryngology clinic reporting nasal obstruction, rhinorrhoea, and postnasal drip over a period of nine to twelve months. As a fly-in, fly-out worker in regional Australia, he travelled to work on an aeroplane every two weeks. He described severe right-sided facial and mastoid tip pain on flying and associated intermittent hearing loss and tinnitus aggravated by these trips. Examination revealed a dull and immobile right tympanic membrane with the remainder of the examination being unremarkable.

**Figure 1:**
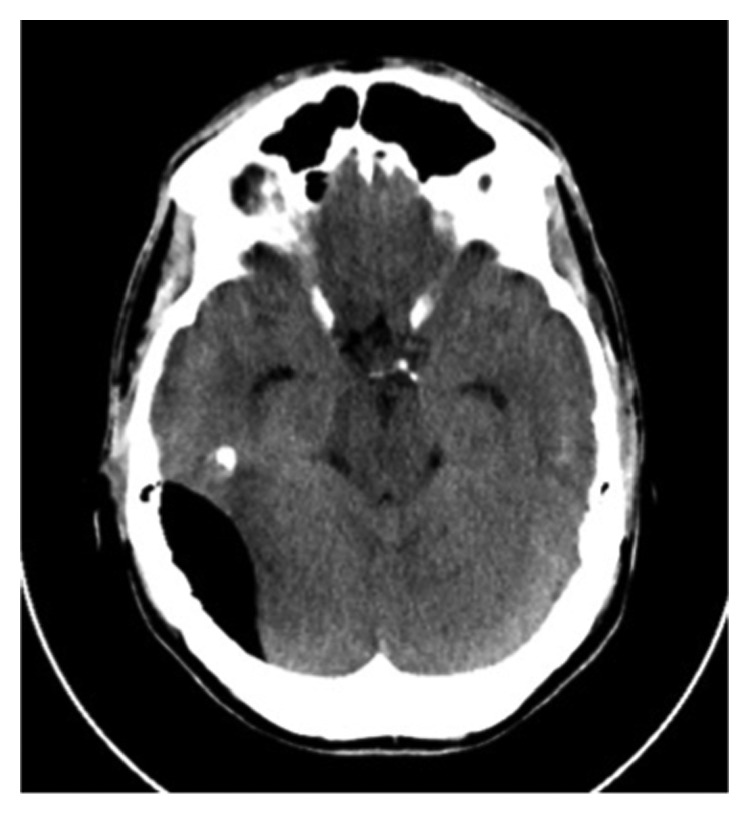
CT head demonstrating right-sided pneumocephalus.

**Figure 2:**
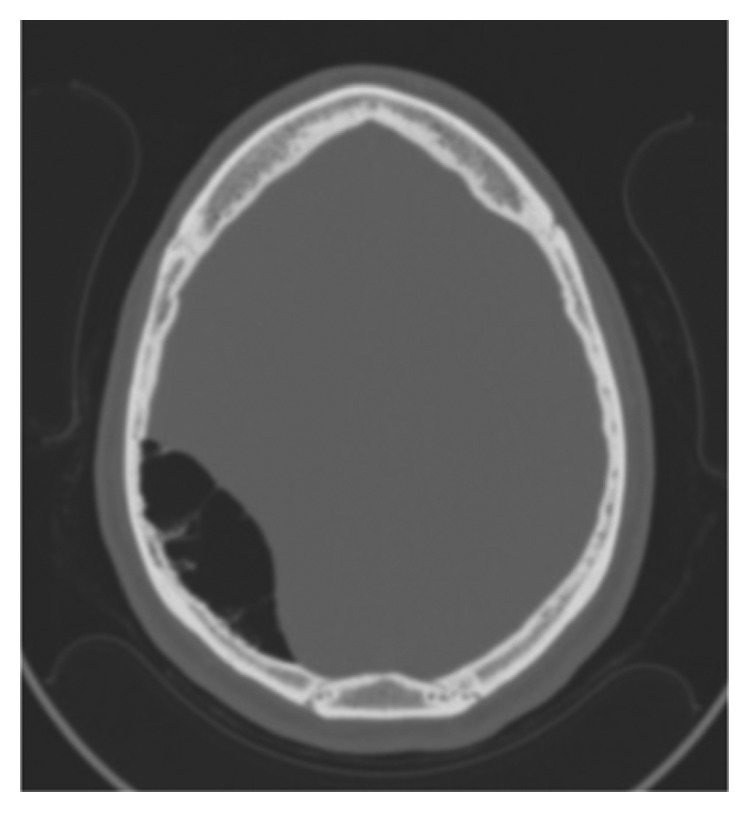
CT head in bone window demonstrating pneumocephalus.

**Figure 3:**
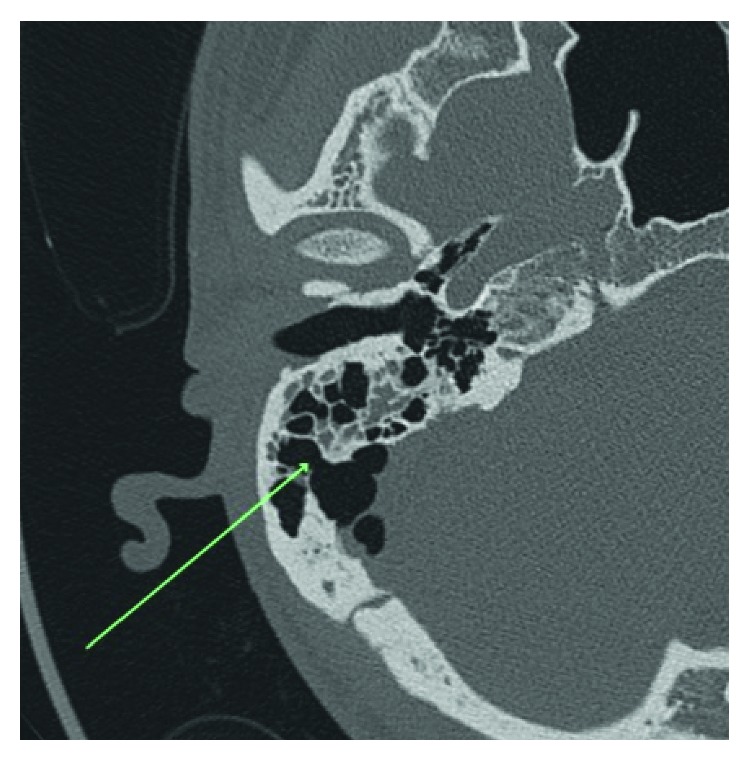
Axial view of CT head demonstrating mastoid defect and pneumocephalus.

**Figure 4:**
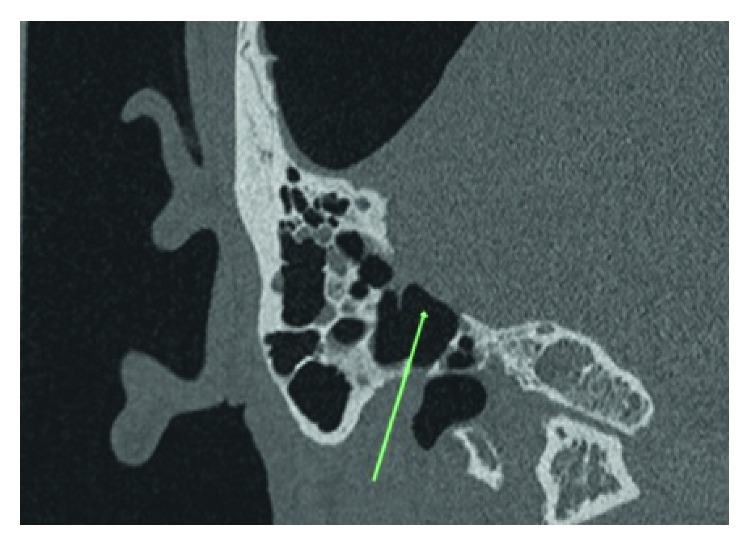
Mastoid defect noted on coronal view.

**Figure 5:**
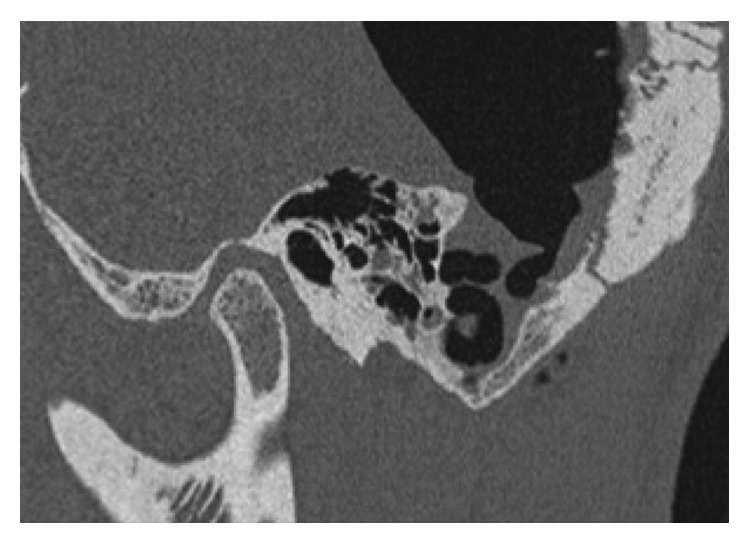
Sagittal CT view.

Further investigation with a computerised tomography scan of the temporal bones was delayed by the patient's work schedule and performed only a month later. Imaging of the right temporal bone demonstrated partial opacification of air cells in the well-pneumatised mastoid process with a multiloculate 26 × 24 × 23 mm air-containing pneumocele centred at the inferomedial margin of the mastoid process. The air was extending via the dehiscent sigmoid venous plate into the groove of the sigmoid venous sinus and inferomedially through a 6 mm defect at the junction of the mastoid process and periphery of the squamous portion of the occipital bone into a subtemporal position. Extending superiorly from this pneumatocele, a very large ovoid extradural air collection was present, measuring 88 × 88 × 40 mm which appeared longstanding. This pneumatocele was also associated with irregular scalloping of the inner table of the skull vault and superior septa, several of which were calcified. There was no air in the carotid canal, jugular bulb, or carotid sheath. The middle ear was clear, the tegmen was intact, and the bony labyrinth was normal. Imaging of the brain showed marked compression of the posterior aspect of the right cerebral hemisphere, principally the parietal lobe. There was subfalcine herniation with deviation of the ventricular midline to the left and mild dilatation of the temporal horns of the lateral ventricles (Figures 1–5).

The patient was directed to the nearest tertiary hospital for otolaryngological and neurosurgical review. The patients' symptoms were unchanged from the initial presentation. On examination, the right tympanic membrane was bulging. No neurological deficits were evident but there was prominent subcutaneous emphysema noted overlying the right mastoid tip.

After appropriate discussion and consent, the patient underwent a tympanostomy tube insertion under local anaesthetic in order to avoid theoretically exacerbating the pneumatocele on anaesthetic induction and positive pressure ventilation. Interestingly, during the tympanostomy tube placement, the patient reported right-sided head pain. General anaesthesia was induced, and the mastoid portion of the surgery was performed. A mastoid cortical bone flap was raised and a mastoidectomy was performed, with identification of the tegmen, sigmoid sinus, lateral and superior semicircular canals, and antrum. The facial nerve was not exposed in the mastoid segment. The mastoid was found to be hyperpneumatised and a fistula was identified from the mastoid air cells to the extradural space at the sinodural angle, with air bubbling noted. There was dehiscence of the posterior fossa dural plate with chronic mucosal disease present adjacent to the dehiscent dura, suggestive of a chronic disease process. However, cerebrospinal fluid was not encountered. The aditus was obliterated with bone wax, and all air cells were obliterated with fat harvested from the left iliac fossa. Closure was performed with fibrin sealant and fascia, the mastoid cortical bone flap was replaced, and the soft tissues were closed in layers. A routine mastoidectomy head bandage was applied.

The patient had an uncomplicated postoperative course and was discharged on postoperative day five. Serial computerised tomography scans at 2 weeks and 4 weeks postoperatively showed stepwise resolution of a small collection in the surgical bed with a reducing mass effect and resolution of the midline shift. At 4 weeks, no pneumocephalus was identified. Encephalomalacia in the posterior right temporal lobe and ex vacuo phenomenon in the occipital horn of the right ventricle were evident. The patient's otological and pain symptoms subsequently resolved. Symptoms and signs of chronic rhinosinusitis were present, and medical management was commenced. The patient subsequently flew to work three months postoperatively.

## 3. Discussion

Lecat first described pneumocephalus in 1741, with Jelsma the first to report a case of spontaneous pneumocephalus in 1954. In his paper “Cranial Aerocele,” [1] Jelsma classified the proposed aetiologies of intracranial and extracranial free gas, including otological sources through the tegmen tympani, the ‘inner mastoid cells' and the ‘outer mastoid cells'. Since this first description, case reports and limited case series have described the phenomenon of spontaneous otogenic pneumocephalus. Patients with spontaneous otogenic pneumocephalus present with heterogenous symptoms, ranging from vague otological symptoms to critical acute neurological events.

Contributory factors to the development of spontaneous otogenic penumocephalus have been postulated to be hyperpneumatisation of the mastoid [2–4], idiopathic defects in the tegmen tympani or mastoid air cells [5] or in the regions of fusion planes between parts of the temporal bone [6], low intracranial pressure or increased middle ear pressure [5], as well as forceful and repetitive nose blowing and Valsalva manoeuvre [4, 7, 8].

In the case described above, a presumed contributing factor was the repetitive barotrauma associated with frequent plane travel to and from the patient's place of employment. Three similar cases have been previously described. Benson et al. [9] describe the case of a 60-year-old who was demonstrated to have intraventricular air on a CT to investigate presyncope. He described ‘sudden onset of a severe rushing sound in both ears' on landing at the conclusion of a flight three months earlier. Multiple small defects in the middle cranial fossa floor over the anterior petrous bone and mastoid air cells were identified and repaired using a middle cranial fossa approach. Wilkinson et al. [1] report a case of a 60-year-old male who developed unilateral tinnitus, aural fullness, and hearing loss after air travel. A unilateral effusion and conductive hearing loss was noted, imaging demonstrated intraventricular air, and a midcranial fossa approach and repair of the tegmen defects was performed. Hage et al. [10] report a case of a 51-year-old male with a two-month history of tinnitus, who developed a rapid neurological decline on ascension to 1000 m in an aeroplane, which improved on descent. Imaging revealed right temporal intraparenchymal, right frontal subdural, and bilateral frontal horn intraventricular air. A right subtemporal craniotomy approach identified a dural defect at the upper aspect of the petrous temporal bone which was repaired.

The aims of surgical management are decompression of the pneumocephalocele and definitive closure of the tegmen or mastoid defect. This can be achieved via a craniotomy or transmastoid approach. In the case described, the source of the pneumocephalus was the inferomedial margin of the mastoid air cells, and the transmastoid approach was ideal. All mastoid air cells were obliterated, with some authors electing to obliterate the mastoid [4, 7] and others closing the defect with a transmastoid approach without obliteration [6, 11].
